# Spray-Dried Plasma Improves Body Weight, Intestinal Barrier Function, and Tibia Strength during Experimental Constant Heat Stress Conditions

**DOI:** 10.3390/ani11082213

**Published:** 2021-07-26

**Authors:** Jared Ruff, Thaina L. Barros, Joy Campbell, Ricardo González-Esquerra, Christine N. Vuong, Sami Dridi, Elizabeth S. Greene, Xochitl Hernandez-Velasco, Billy M. Hargis, Guillermo Tellez-Isaias

**Affiliations:** 1 Department of Poultry Science, University of Arkansas, Fayetteville, AR 72701, USA; jaruff@uark.edu (J.R.); tbarros@uark.edu (T.L.B.); vuong@uark.edu (C.N.V.); dridi@uark.edu (S.D.); esgreene@uark.edu (E.S.G.); bhargis@uark.edu (B.M.H.); gtellez@uark.edu (G.T.-I.); 2 APC, LLC, Ankeny, IA 50021, USA; gesquerra@hotmail.com; 3 Department of Avian Medicine, College of Veterinary Medicine, National Autonomous University of Mexico, Mexico City 04510, Mexico; xochitl_h@yahoo.com

**Keywords:** broiler chickens, tibia strength, heat stress, leaky gut, spray-dried plasma

## Abstract

**Simple Summary:**

Broilers are especially heat sensitive because of the absence of sweat glands and their elevated metabolism. Under commercial conditions, extremely high temperatures (heat stress) reduce their performance. This research aimed to assess spray-dried feeding plasma (SDP) during constant heat stress (HS) on the performance, intestinal permeability, and bone strength in broilers. Chickens fed with a diet supplemented with SDP increased both their body weight and body weight gain compared to the HS control group. At the end of the study (d 42 of age), chickens fed with SDP significantly alleviated the increased gut leakage induced by HS and showed a significant increase in tibia strength compared with control HS chickens. The results in the present study suggest SDP mends gut integrity, hence reducing chronic systemic inflammation.

**Abstract:**

The aim of this study was to see how spray-dried plasma (SDP) supplementation affected broiler chicken performance, intestinal permeability, and bone strength during persistent heat stress. One-day-old chicks (*n* = 480) were randomly assigned into twelve environmental corrals; four thermoneutral (TN-negative control, maintained at 24 °C from d 21–42); four heat stress (HS, exposed to 35 °C from d 21–42); and four heat stress treated with 2% SDP in the feed until d 28 followed by 1% SDP until d 42 (HS-SDP). The performance and serum levels of fluorescein isothiocyanate-dextran (FITC-d) were evaluated at d 21, 28, 35, and 42. The tibias strength was evaluated on d 21 and 42. The increment in chicken temperature (*p* < 0.05) was observed two h following the increase in environmental temperature in both HS groups and was associated with decreased performance parameters compared with the TN group. At d 42 of age, the chickens exposed to HS had an impaired gut permeability and decreased tibia strength compared to the TN group (*p* < 0.05). However, partially feeding SDP mitigated these adverse effects significantly. These findings imply that using SDP strategically during stressful times, such as prolonged heat stress, may help mitigate its negative consequences.

## 1. Introduction

Since 2001, there has been a documented increase in hot temperatures, and climate trends are anticipated to continue [1]. These increments in temperatures will serve as a severe environmental stress concern to plants and animals [2,3]. However, broilers are especially heat sensitive because of the absence of sweat glands as well as their elevated metabolism [4,5]. According to a recent study, heat stress costs the US broiler poultry sector over a hundred million dollars per year [6].

Spray-dried plasma (SDP) has been used in monogastric and ruminants in the presence or absence of antibiotic growth promoters (AGP) [7]. Immunoglobulins, albumin, growth factors, and biologically active peptides are examples of functional proteins present in SDP that modulate the immune response and improve intestinal health [8,9,10,11]. Therefore, the continued understanding of SDP’s benefits on gut integrity in poultry during different types of stress and feeding regimes will help the poultry industry better utilize SDP as a management tool. Recently, we published that continuous heat stress is a reliable model to induce intestinal inflammation [12]. Hence, we postulated that the immunological properties of SDP could reduce gastrointestinal leakage in chicks under continuous experimental heat conditions. Leaky gut has been associated with multiple organ failure and local and systemic inflammation [13]. Several poultry models have confirmed that fluorescein isothiocyanate-dextran is a reliable biomarker to measure intestinal permeability [14]. The purpose of this investigation was to assess feeding SDP during constant heat stress on performance, intestinal permeability, and bone strength in broilers.

## 2. Materials and Methods

### 2.1. Ethics

The Institutional Animal Care and Use Committee (IACUC) at the University of Arkansas, Fayetteville, approved all animal handling methods. This study was approved by the IACUC under protocol # 18030.

### 2.2. Spray-Dried Plasma

Spray-dried plasma (Appetein, APC, LLC, Ankeny, IA 50021, USA) is a feed ingredient widely used in animal diets; it is effective in helping animals mitigate the consequences of the most stressful growth phases. The composition of the SDP is summarized in Table 1.

### 2.3. Animals and Diets

A commercial hatchery provided one-day-old Cobb 500 male broiler chicks (*n* = 480). Chickens were vaccinated with a coccidia vaccine (Coccivac^®^-B52 Merck Animal Health, De Soto, KS 66018). Chickens were neck tagged and randomly allocated to twelve environmental rooms: four thermoneutral (TN); four heat stress (HS); and four heat stress supplemented with 2% SDP in the feed until d 28 followed by 1% SDP until d 42 (HS-SDP). The diets employed in this study were adjusted to match breeder guidelines [15]. No growth promoters were included in the diets. Diets provided an adequate supply of nutrients, and the proportions of the feed ingredients used were adjusted to the nutrient contribution of SDP so that diets with similar nutrient profiles were fed across treatments (Table 2).

### 2.4. Experimental Design

This study was conducted at the Poultry Environmental Research Laboratory (PERL) at the University of Arkansas. In this facility, there are 12 environmental rooms; each has its own air conditioner unit with a thermostat. In the present study, each environmental room was divided into two-floor corrals (150 cm × 300 cm), with feeders and automatic watering systems (*n* = 8 repeats per treatment; *n* = 20 birds/corral for *n* = 160 chickens/treatment). From d 1 to 21 in all rooms, temperature and light were controlled to imitate commercial circumstances, with a steady decrease in temperature from 32 to 24 °C and relative humidity at 55 ± 5%. The TN group was kept at 24 °C from d 21 to 42, whereas the heat stress experimental groups were exposed to 35 °C. Temperature and relative humidity were monitored three times a day. On d 18, a chicken from each pen was chosen at random to have a Thermochron temperature logger inserted into its beak (iButton, DS1922L, Embedded Data Systems, Lawrenceburg, KY, USA). As described by Flees et al. [16], the devices remained in the gizzard for body temperature measurement. The chickens’ body temperatures were recorded every minute for the first two h after starting the heat stress and then every hour after that. Individual body weight (BW) and body weight gain (BWG) were recorded from each experimental replicate for performance. Feed intake (FI) and feed conversion rate (FCR) were evaluated per replicate (*n* = 8). Performance parameters were collected at d 11, 22, 28, 35, and 42.

### 2.5. Serum Fluorescein Isothiocyanate-Dextran Determination

On d 21, 28, 35, and 42, two chicks were chosen at random from each pen (*n* = 16) and gavaged with fluorescein isothiocyanate-dextran at a dose of 8.32 mg/kg body weight (FITC-d, MW 3–5 KDa; Sigma-Aldrich Co., St. Louis, MO, USA). Chickens were euthanized by CO_2_ exposure an hour after receiving FITC-d. Blood samples were drawn from the femoral vein and centrifuged (1000× *g* for fifteen minutes) to separate the serum. Baxter et al. [17] stated that serum levels of FITC-d were utilized as a biomarker to assess leaky gut.

### 2.6. Bone Parameters

The left tibia from each sampled chicken (*n* = 16) was removed to assess break strength (kg) and total ash on d 21 and 42, as described by Gautier et al. [18].

### 2.7. Statistical Analysis

Results were evaluated utilizing the PROC general linear models system of statistical analysis software [19]. An analysis of variance was conducted to detect differences among dietary treatments. Treatment was the independent variable. Dependent variables were body weight, average gain, feed intake, feed conversion, bone-breaking strength, and intestinal permeability. The mean values of all dietary regimens were calculated using the least squares (marginal) means (LSMEANS). If treatment effects were found, least square means were used to differentiate the groups by the requested p-values for differences (PDIFF) option in SAS (Statistical Analysis System). Significance used to assess differences was declared at *p* < 0.05 unless otherwise reported.

## 3. Results

**Figure 1:**
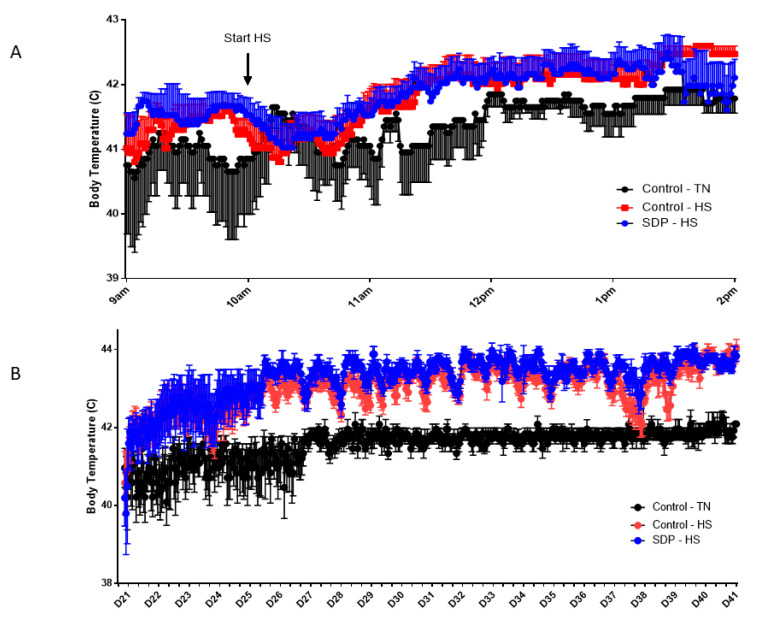
Evaluation of core body temperature of broiler chickens under thermoneutral (TN) conditions compared with broiler chickens during continuous heat stress (HS) without spray-dried plasma (SDP) or with dietary supplementation of SDP during acute (**A**) and chronic (**B**) heat stress (*p* < 0.001).

Figure 1 displays the outcomes of the evaluation of the core body temperature of chicks supplemented with SDP during continuous acute and chronic heat stress. Just two h after initiating heat stress in the corrals of the experimental HS groups, the body core temperature of the chickens was considerably higher than that of the control TN group and persisted through the termination of the trial, with severe repercussions in performance parameters (Table 3 and Table 4).

The results of the assessment of BW and BWG in chicks supplemented with SDP during continuous heat stress are described in Table 3. Birds that received SDP during the first eleven days significantly (*p* < 0.05) increased BW by 10% (~22 g). As expected, heat stress significantly reduced BW at d 28. However, chickens fed with 2% SDP increased BW by 6% (~75 g) compared to the heat stress control group. A similar trend was observed at d 42, where chickens under continuous heat stress had a significantly reduced BW at d 42. In contrast, chickens fed SDP early and during heat stress increased BW by 8% (~135 g) compared to heat stress control chickens (Table 3). These significant increments in BW at d 28, 35, and 42 in chickens fed with 2% SDP under heat stress were also reflected in BWG during the same periods of evaluation compared to heat stress control chickens (Table 3).

Table 4 shows the evaluation of FI and FCR during continuous heat stress. In the present study, chickens in both experimental groups that were exposed to constant heat stress showed a significantly reduced feed intake through d 42 when compared with TN control chickens. Similarly, heat stress significantly increased feed conversion from d 0–42. However, feeding plasma did not mitigate the increase in feed conversion resulting from heat stress (Table 4).

Nevertheless, chickens consuming plasma numerically mitigated some of the reduction in feed intake resulting from heat stress.

The assessment of serum FITC-d in birds supplemented with SDP during continuous heat stress is summarized in Table 5. Interestingly, before initiating heat stress, a significant variation was observed between treatments on d 21. However, on d 35, both experimental groups receiving continuous HS showed increased levels of FITC-d in the serum, compared with the control TN group. Nevertheless, at the termination of the trial on d 42, chickens fed with SDP during continuous heat stress significantly alleviated the increase in gut permeability (Table 5).

The findings of bone mineralization in birds supplemented with SDP during continuous heat stress are presented in Table 6. On d 21, no changes in the treatment groups were noticed. However, when compared to control TN chickens on d 42, the constant heat exposure resulted in a considerable decline in tibia strength. When chickens were fed SDP and then subjected to heat stress, they demonstrated a substantial increase in tibia strength when compared to control heat stress chicks. Furthermore, heat-stressed birds exhibited a larger percentage of tibia ash than thermoneutral control birds (Table 6).

## 4. Discussion

Poultry is exceptionally susceptible to heat due to the absence of sweat glands and their tremendous metabolic rate [5]. Unfortunately, the pressure of an exceeded growth rate and feed efficiency is not accompanied by an increase in cardiovascular and respiratory functionality [20]. As a result, heat stress has become one of the most severe economic challenges for the poultry sector [6] since birds absorb more heat than is dissipated [21]. To compensate for the absence of sweat glands, birds have evolved alternative mechanisms for maintaining physiological homeostasis and regulating core temperature, including convective cooling, evaporation, and radiation [20]. The harmful consequences of heat stress can vary from heat exhaustion to cellular, tissue, and organ impairment. Severe or chronic heat stress can cause heatstroke and death [21]. Under commercial conditions, HS reduces the poultry’s performance [4,16]. Perhaps one of the most susceptible tissues to heat stress is the gastrointestinal tract, as it decreases tight junction protein gene expression, leading to an increase in permeability and chronic systemic inflammation [22,23,24]. In agreement with previous publications, heat stress hurt the performance of broilers [12]. However, in the present study, the inclusion of SDP led to improvements in BW and BWG and numerically alleviated the reduction in feed intake compared to heat stress birds not supplemented with SDP. The decrease in feed intake observed in both heat stress experimental groups affected performance parameters.

Interestingly, the addition of SDP reduced serum concentrations of FITC-d. Due to the small molecular size of FITC-d (3–5 kDa), the molecule is not absorbed by the gastrointestinal tract under normal conditions. Nonetheless, regardless of its source, any stress induces oxidative stress in the enterocytes and inflammation, causing down-regulation of the tight junction proteins and leading to an increased permeability [13]. Hence, FITC-d has become a reliable and essential biomarker to evaluate leaky gut in poultry [14].

The improvement of intestinal barrier function has also been reported with the dietary supplementation of spray-dried chicken plasma in weaning piglets [25]. In another study, the significant physiological and performance parameters observed in pigs supplemented with SDP and subjected to heat stress were linked to the enhancement of gut barrier integrity, antioxidant activity, and immune modulation [26]. This is the first study confirming the reduction of FITC-d in the serum of SDP-supplemented heat stress chickens. This finding agrees with previous publications and suggests an improvement of intestinal barrier function and gut integrity, hence, reducing chronic systemic inflammation [7,27]. These findings may help to explain why chickens exposed to heat and supplemented with SDP performed better than chickens that are not exposed to heat.

On d 42, heat stress significantly reduced bone strength, which is consistent with several studies confirming that inflammation reduces bone mineralization, healing, and regeneration [28,29,30]. Nevertheless, chickens under chronic and continuous heat stress supplemented with SDP in the feed showed substantial bone strength than control HS chickens. Recent studies in weaning piglets have shown that the dietary supplementation of SDP also increases BW and reduces feed conversion by enhancing intestinal digestive function and regulating specific microbiota in the gut [31]. These effects have been associated with reducing the animals to microbial or dietary antigens and anti-inflammatory properties of SDP [9,27,32,33]. The improvements reported with SDP in commercial poultry operations are more significant than cleaner research settings [7]. Furthermore, several studies have confirmed that SDP reduces the damage of enteropathogens [32,33,34] and improves the digestibility of amino acids and microbiota diversity [35,36]. These results suggest that SDP is a safe alternative to AGPs, particularly under stressful conditions [8,34,37,38,39].

In poultry nutrition, protein products play a critical role in the birds’ biology and performance. Several investigators have shown that feeding SDP to broiler chicks during their first ten days improves their gastrointestinal physiology and performance through reducing intestinal inflammation and immune regulation [11,35,40]. Furthermore, it is essential to know the protein sources for the formulation of the diets in order to reduce the presence of anti-nutritional factors and maintain gut health [13].

## 5. Conclusions

Heat stress reduced BW, feed intake, and bone strength, and increased feed conversion and gut permeability. However, feeding plasma during continuous HS mitigated the effects of HS on BW, FI, gut permeability, and bone strength to a certain extent. These findings suggest that strategic use of SDP during times of stress, such as prolonged HS, may mitigate its adverse effects on performance, intestinal permeability, and bone strength reduction. Further studies to evaluate SDP’s effect on cyclic heat stress and other inflammatory biomarkers are presently undergoing evaluation.

## Figures and Tables

**Table 1:** Supplier reported composition of spray-dried plasma.

Item	Spray-Dried Plasma ^1^
Dry matter, %	92
ME, kcal/kg	3532
Ash, %	10
Ca, %	0.15
P, %	1.30
Na, %	2.20
Cl, %	1.10
K, %	0.30
CP and AA	
CP, %	77.0
Arg, %	4.60
Cys, %	2.40
His, %	2.70
Ile, %	2.80
Leu, %	7.60
Lys, %	6.60
Met, %	0.60
Phe, %	4.50
Thr, %	4.20
Trp, %	1.40
Tyr, %	3.50
Val, %	5.20

^1^ Spray-dried plasma product name: Appetein, APC, LLC, Ankeny, IA 50021, USA.

**Table 2:** Control corn–soybean diet’s ingredient mix and nutrient content, or control corn–soybean diet supplemented with spray-dried plasma (SDP) used on an as-is basis.

Item	Starter Control Diet	Starter SDP Diet	Grower Control Diet	Grower SDP Diet	Finisher Control Diet	Finisher SDP Diet
Ingredients (%)						
Corn 9-14-18	51.80	54.38	57.81	60.39	59.64	60.93
SBM (45.16%)	37.66	33.96	31.62	27.92	27.23	25.38
DDGS 8.1% EE	4.00	4.00	4.00	4.00	6.00	6.00
Poultry fat	3.24	2.55	3.44	2.76	4.38	4.04
SDP	-	2.00	-	2.00	-	1.00
Limestone	1.08	1.18	1.06	1.15	1.03	1.08
Phosphate of dicalcium	1.01	0.89	0.88	0.76	0.64	0.58
Sodium chloride	0.35	0.27	0.35	0.23	0.31	0.24
DL-Methionine	0.29	0.23	0.25	0.22	0.22	0.21
L-Lysine Hydrochloride	0.12	0.10	0.13	0.10	0.12	0.10
Waldroup TM Mix	0.10	0.10	0.10	0.10	0.10	0.10
Tyson 2x Broiler Vit	0.10	0.08	0.10	0.09	0.10	0.10
L-threonine	0.08	0.08	0.09	0.08	0.09	0.07
Choline chloride (60%)	0.06	0.07	0.06	0.06	0.05	0.06
Sodium bicarbonate	0.04	0.06	0.05	0.06	0.03	0.04
OptiPhos2000 (0.5 lb/ton)	0.025	0.025	0.025	0.025	0.025	0.025
Se Premix (0.06%)	0.020	0.020	0.020	0.020	0.020	0.020
Santoquin	0.019	0.019	0.019	0.019	0.019	0.019
Calculated analysis						
ME (kcal/kg)	3015.00	3015.00	3090.00	3090.00	3175.00	3175.00
Ether extract (%)	5.88	5.25	6.20	5.57	7.28	6.96
Crude protein (%)	22.30	22.30	20.00	20.00	18.70	18.70
Lysine (%)	1.18	1.18	1.05	1.05	0.95	0.95
Methionine (%)	0.59	0.56	0.53	0.50	0.48	0.46
Threonine (%)	0.77	0.77	0.69	0.69	0.65	0.65
Tryptophan (%)	0.25	0.25	0.22	0.22	0.20	0.20
Total calcium (%)	0.90	0.90	0.84	0.84	0.76	0.76
Total phosphorous (%)	0.63	0.59	0.58	0.54	0.53	0.51
Available phosphorus (%)	0.45	0.45	0.42	0.42	0.38	0.38
Sodium (%)	0.20	0.20	0.20	0.20	0.18	0.18
Potassium (%)	1.06	0.99	0.94	0.87	0.87	0.83
Chloride (%)	0.27	0.21	0.28	0.21	0.25	0.22
Magnesium (%)	0.19	0.18	0.18	0.17	0.17	0.17
Copper (%)	19.20	18.71	18.46	17.98	18.85	18.61
Selenium (%)	0.28	0.27	0.27	0.26	0.26	0.26
Linoleic acid (%)	1.01	1.06	1.13	1.18	1.16	1.19

Starter diet from d 0–10; grower diet from d 11–28; and finisher diet from d 28 to 42.

**Table 3:** Evaluation of body weight and body weight gain in broiler chickens under thermoneutral conditions compared with broiler chickens during continuous heat stress (HS) without spray-dried plasma (SDP) or with dietary supplementation of SDP.

Days	Control Thermoneutral	Control HS	SDP-HS	SEM
Body weight				
0 d	41.14	41.45	41.40	0.26
11 d	224.96 ^b^	219.42 ^b^	244.07 ^a^	3.68
22 d	880.05	873.80	895.02	11.86
28 d	1510.20 ^a^	1260.18 ^c^	1334.92 ^b^	17.92
35 d	2283.00 ^a^	1515.53 ^c^	1624.99 ^b^	30.50
42 d	2913.48 ^a^	1714.69 ^c^	1850.46 ^b^	50.64
Body weight gain				
0–11 d	183.63 ^b^	178.09 ^b^	202.74 ^a^	3.68
0–22 d	838.69	832.44	853.66	11.86
0–28 d	1468.94 ^a^	1218.92 ^c^	1293.65 ^b^	17.92
0–35 d	2241.82 ^a^	1474.35 ^c^	1583.81 ^b^	30.50
0–42 d	2872.32 ^a^	1673.53 ^c^	1809.30 ^b^	50.64

Data expressed as least squares means ± SEM of 8 replicates per treatment. ^a–c^ Values within rows with different superscripts are statistically significant.

**Table 4:** Evaluation of feed intake and feed conversion ratio in broiler chickens under thermoneutral conditions compared with broiler chickens during continuous heat stress (HS) without spray-dried plasma (SDP) or with dietary supplementation of SDP.

Days	Control Thermoneutral	Control HS	SDP-HS	SEM
Feed intake				
0–11 d	141.44	135.08	136.02	9.64
0–22 d	1058.90	1029.18	1070.50	32.48
0–28 d	1876.88 ^b^	1668.99 ^c^	1778.73 ^ab^	54.26
0–35 d	3101.35 ^a^	2566.45 ^b^	2713.42 ^b^	63.41
0–42 d	4239.76 ^a^	3157.23 ^b^	3332.08 ^b^	92.49
Feed conversion				
0–11 d	0.774	0.779	0.672	0.067
0–22 d	1.264	1.233	1.253	0.023
0–28 d	1.280 ^b^	1.368 ^a^	1.375 ^a^	0.023
0–35 d	1.383 ^b^	1.730 ^a^	1.735 ^a^	0.052
0–42 d	1.475 ^b^	1.881 ^a^	1.878 ^a^	0.054

Data expressed as least squares means ± SEM of 8 replications per treatment. ^a–c^ Values within rows with different superscripts are statistically significant.

**Table 5:** Assessment of FITC-d in the serum of chickens under thermoneutral conditions compared with broiler chickens during continuous heat stress (HS) without spray-dried plasma (SDP) or with dietary supplementation of SDP.

Serum FITC-d (ng/mL)	Control Thermoneutral	Control HS	SDP-HS	SEM
Day 21	231.37 ^A^	157.08 ^C^	192.10 ^B^	12.02
Day 28	240.74	247.67	251.13	10.84
Day 35	177.65 ^b^	235.79 ^a^	248.41 ^a^	13.49
Day 42	218.55 ^C^	312.60 ^A^	276.64 ^B^	15.00

Data expressed as least squares means ± SEM. ^a,b^ at *p* < 0.05 and ^A–C^  *p* < 0.10, values in rows with distinct superscripts are statistically significant.

**Table 6:** Evaluation of bone mineralization in chickens under thermoneutral conditions compared with broiler chickens during continuous heat stress (HS) without spray-dried plasma (SDP) or with dietary supplementation of SDP.

Days	Control Thermoneutral	Control HS	SDP-HS	SEM
Tibia strength (kg)				
Day 21	17.93	18.79	16.86	0.99
Day 42	37.71 ^A^	24.37 ^C^	29.74 ^B^	2.01
Total ash from the tibia (%)				
Day 21	54.07	54.57	53.23	0.52
Day 42	54.78 ^b^	56.33 ^a^	56.89 ^a^	0.32

Data expressed as least squares means ± SEM. ^a,b^ at *p* < 0.05 and ^A–C^ at *p* < 0.10, values in rows with distinct superscripts are statistically significant.

## Data Availability

The raw data supporting the conclusions of this article will be made available by the authors, without undue reservation.
